# Crystal structure and Hirshfeld surface analysis of 1,3-bis­{2,2-di­chloro-1-[(*E*)-phenyl­diazen­yl]ethen­yl}benzene

**DOI:** 10.1107/S2056989021007192

**Published:** 2021-07-16

**Authors:** Namiq Q. Shikhaliyev, Zeliha Atioğlu, Mehmet Akkurt, Nigar E. Ahmadova, Rizvan K. Askerov, Ajaya Bhattarai

**Affiliations:** a Organic Chemistry Department, Baku State University, Z. Khalilov str. 23, AZ 1148 Baku, Azerbaijan; b Department of Aircraft Electrics and Electronics, School of Applied Sciences, Cappadocia University, Mustafapaşa, 50420 Ürgüp, Nevşehir, Turkey; c Department of Physics, Faculty of Sciences, Erciyes University, 38039 Kayseri, Turkey; d Department of Chemistry, M.M.A.M.C (Tribhuvan University) Biratnagar, Nepal

**Keywords:** crystal structure, C—H⋯π, C—Cl⋯π, Cl⋯Cl, Cl⋯H inter­actions, Hirshfeld surface analysis

## Abstract

In the mol­ecule of the title compound, C_22_H_14_Cl_4_N_4_, the central benzene ring makes dihedral angles of 77.03 (9) and 81.42 (9)° with the two approximately planar 2,2-di­chloro-1-[(*E*)-phenyl­diazen­yl]vinyl groups. In the crystal, mol­ecules are linked by C—H⋯π, C—Cl⋯π, Cl⋯Cl and Cl⋯H inter­actions, forming a three-dimensional network. The Hirshfeld surface analysis indicates that the most important contributions to the crystal packing are from H⋯H (30.4%), C⋯H/H⋯C (20.4%), Cl⋯H/H⋯Cl (19.4%), Cl⋯Cl (7.8%) and Cl⋯C/C⋯Cl (7.3%) inter­actions.

## Abstract 2

In the crystal, mol­ecules of the title compound are connected through C—H⋯π, C—Cl⋯π, Cl⋯Cl and Cl⋯H inter­actions, generating a three-dimensional network.

## Chemical context   

Azodyes and related hydrazones are of inter­est for synthetic organic chemistry, coordination chemistry, medicinal and material chemistry because of their important physical and biological properties (Mahmoudi *et al.*, 2016 ▸, 2017*a* ▸,*b* ▸,*c* ▸, 2018*a* ▸,*b* ▸, 2019 ▸; Viswanathan *et al.*, 2019 ▸). For this reason, diverse new synthetic procedures have been developed for their efficient and versatile synthesis (Gurbanov *et al.*, 2017 ▸, 2018*a* ▸,*b* ▸; Ma *et al.*, 2017*a* ▸,*b* ▸). Moreover, azo/hydrazone ligands can also be used as starting materials in the synthesis of coordination and supra­molecular compounds (Ma *et al.*, 2020 ▸, 2021 ▸; Mahmudov *et al.*, 2013 ▸; Sutradhar *et al.*, 2015 ▸, 2016 ▸), and as building blocks in the construction of 1D, 2D or 3D networks owing to their non-covalent bond-donating and acceptor capabilities (Gurbanov *et al.*, 2020*a* ▸; Kopylovich *et al.*, 2011*a* ▸,*b* ▸; Asgarova *et al.*, 2019 ▸). In fact, inclusion of suitable substituents to azo/hydrazone ligands can improve their functional properties and the catalytic or biological activity of the corresponding coordination compounds (Mizar *et al.*, 2012 ▸; Gurbanov *et al.*, 2020*b* ▸; Karmakar *et al.*, 2017 ▸; Khalilov *et al.*, 2011 ▸, 2018*a* ▸,*b* ▸; Mac Leod *et al.*, 2012 ▸; Maharramov *et al.*, 2019 ▸; Shikhaliyev *et al.*, 2019 ▸; Shixaliyev *et al.*, 2014 ▸). Thus, the attachment of halogen-containing substituents to azo/hydrazone compounds can improve their functional properties *via* inter­molecular halogen bonding. In order to continue our work in this perspective, we have synthesized a new halogen­ated bis-azo ligand, 1,3-bis­{2,2-di­chloro-1-[(*E*)-phenyl­diazen­yl]ethen­yl}benzene, which is able to provide multiple inter­molecular non-covalent inter­actions. 
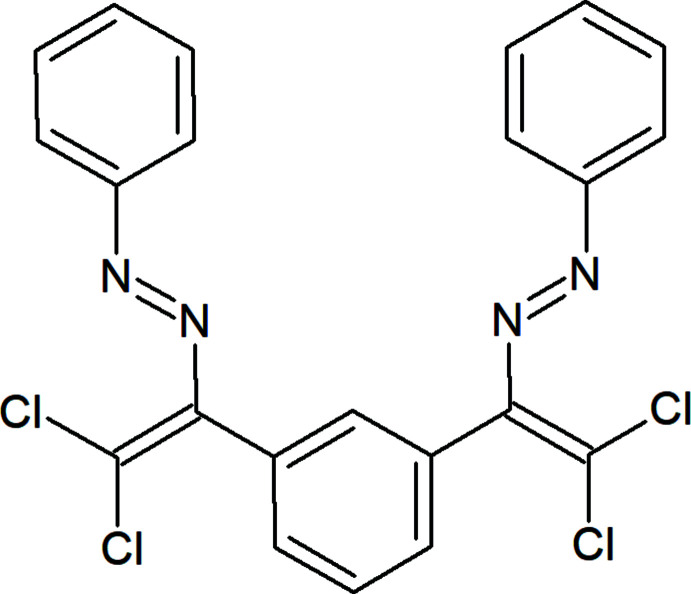


## Structural commentary   

**Figure 1:**
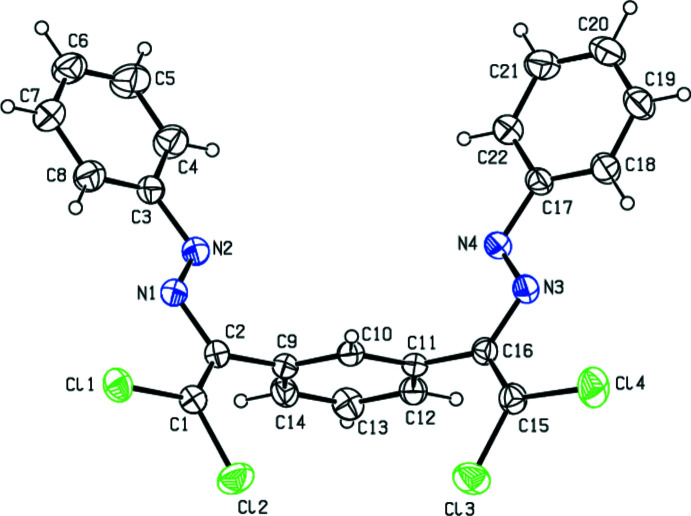
The title mol­ecule with the labelling scheme and 30% probability ellipsoids.

The mol­ecule of the title compound consists of three nearly planar fragments: the central benzene ring and the two attached 2,2-di­chloro-1-[(*E*)-phenyl­diazen­yl]vinyl groups, Cl1–C8 and Cl3–C22 (Fig. 1 ▸), the largest deviations from the least-squares planes of these side groups being 0.060 (1) and 0.083 (3) Å for Cl2 and C18, respectively. These groups are nearly perpendicular to the central benzene ring, subtending dihedral angles of 77.03 (9) and 81.42 (9)°, respectively, with this ring. All bond dimensions within the mol­ecule are typical of such type of compounds (Allen *et al.*, 1987 ▸).

## Supra­molecular features   

**Figure 2:**
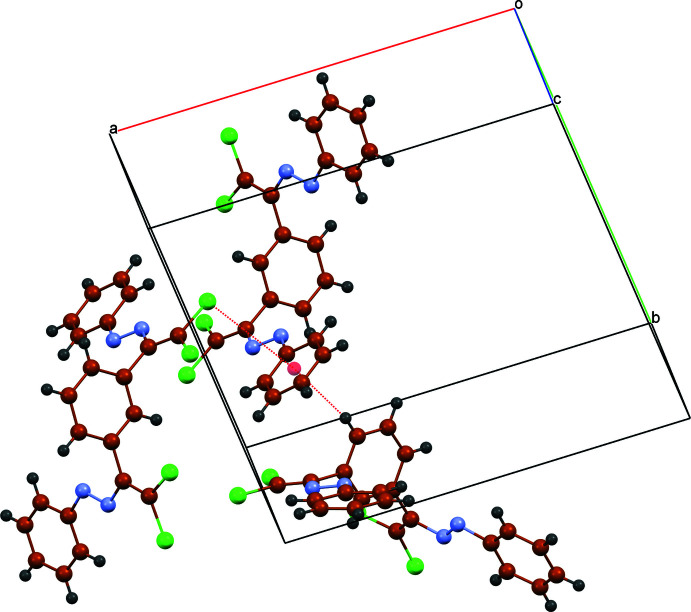
A fragment of the mol­ecular packing showing the C—H⋯π and C—Cl⋯π inter­actions.

In the crystal, mol­ecules are linked by C—H⋯π (Table 1 ▸) and C—Cl⋯π inter­actions [C15—Cl4⋯*Cg*3^ii^; Cl4⋯*Cg*3^ii^ = 3.9572 (15); C15⋯*Cg*3^ii^ = 4.381 (3) Å; C15—Cl4⋯*Cg*3^ii^ = 92.60 (10)°; symmetry code: (ii) 2 − *x*, 1 − *y*, 1 − *z*] involving the terminal C17–C22 phenyl ring (*Cg*3). Besides this, there are the Cl⋯Cl and Cl⋯H contacts, which contribute to a three-dimensional network (Table 2 ▸, Figs. 2 ▸ and 3 ▸).

## Hirshfeld surface analysis   

**Figure 4:**
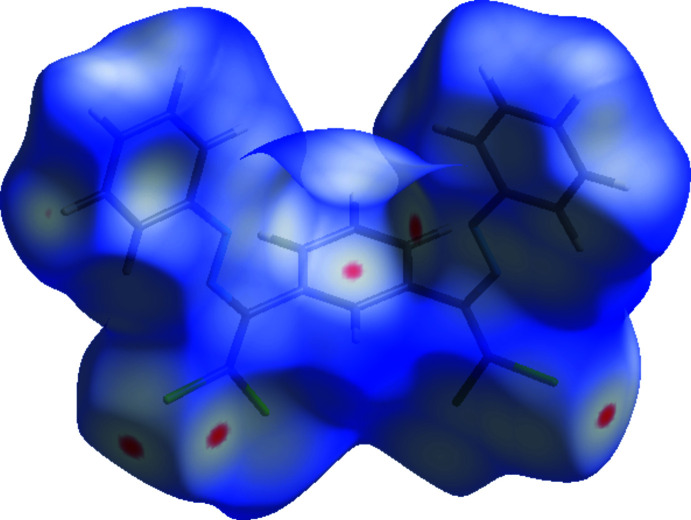
View of the three-dimensional Hirshfeld surface of the title compound plotted over *d*_norm_ in the range −0.0453 to 1.4337 a.u.

**Figure 5:**
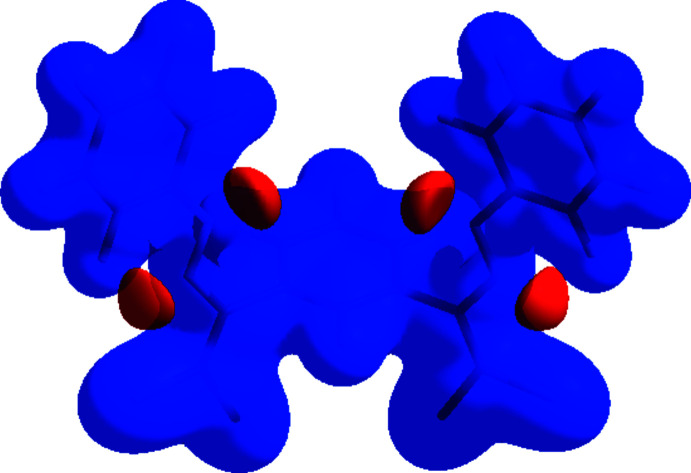
View of the three-dimensional Hirshfeld surface of the title complex plotted over electrostatic potential energy in the range −0.1379 to 0.1988 a.u. using the STO-3G basis set at the Hartree–Fock level of theory. The hydrogen-bond donors and acceptors are viewed as blue and red regions around the atoms corresponding to positive and negative potentials, respectively.

The Hirshfeld surfaces and two-dimensional fingerprint plots were generated using *Crystal Explorer 17.5* (Turner *et al.*, 2017 ▸). Hirshfeld surfaces show inter­molecular inter­actions by different hues and intensities to denote short and long contacts, as well as the intensity of the connections. In Fig. 4 ▸, the 3D Hirshfeld surface of the title mol­ecule is mapped over *d*_norm_ in the range −0.0453 to 1.4337 a.u. The red patches surrounding Cl1, Cl2, Cl3 and Cl4 are caused by the Cl1⋯Cl4, Cl3⋯Cl2 and Cl3⋯H20*A* inter­actions, which play a vital role in the mol­ecular packing of the title compound, and highlight their functions as donors and/or acceptors; they also appear as blue and red regions on the Hirshfeld surface mapped over electrostatic potential (Spackman *et al.*, 2008 ▸) corresponding to positive and negative potentials, as shown in Fig. 5 ▸. The blue regions indicate positive electrostatic potential (hydrogen-bond donors), while the red regions indicate negative electrostatic potential (hydrogen-bond acceptors).

**Figure 6:**
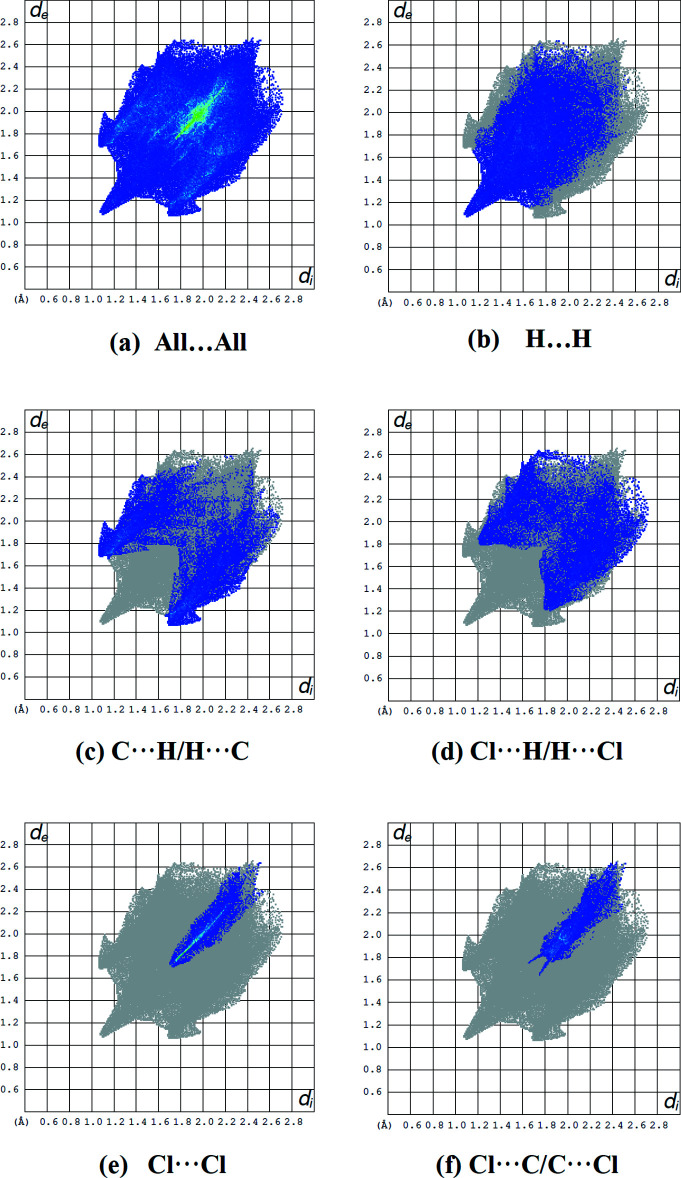
The full two-dimensional fingerprint plots for the title compound, showing (*a*) all inter­actions, and delineated into (*b*) H⋯H, (*c*) C⋯H/H⋯C, (*d*) Cl⋯H/H⋯Cl, (*e*) Cl⋯Cl and (*f*) Cl⋯C/C⋯Cl inter­actions. The *d*_i_ and *d*_e_ values are the closest inter­nal and external distances (in Å) from given points on the Hirshfeld surface.

In Fig. 6 ▸, the overall two-dimensional fingerprint plot for the title compound and those delineated into H⋯H, C⋯H/H⋯C, Cl⋯H/H⋯Cl, Cl⋯Cl and Cl⋯C/C⋯Cl contacts, as well as their relative contributions to the Hirshfeld surface, are shown, while Table 2 ▸ provides data on the distinct inter­molecular contacts. The percentage contributions to the Hirshfeld surfaces from various inter­atomic contacts are: H⋯H (30.4%; Fig. 6 ▸ *b*), C⋯H/H⋯C (20.4%; Fig. 6 ▸ *c*), Cl⋯H/H⋯Cl (19.4%; Fig. 6 ▸ *d*), Cl⋯Cl (7.8%; Fig. 6 ▸ *e*) and Cl⋯C/C⋯Cl (7.3%; Fig. 6 ▸ *f*). Other Cl⋯N/N⋯Cl, N⋯H/H⋯N, C⋯C, N⋯C/C⋯N and N⋯N contacts account for less than 5.9% of Hirshfeld surface mapping and have minimal directional impact on mol­ecular packing (Table 3 ▸).

## Database survey   

A search of Cambridge Crystallographic Database (CSD, version 5.41, update of August 2020; Groom *et al.*, 2016 ▸) revealed a closely related compound, meso-(*E*,*E*)-1,10-[1,2-bis­(4-chloro­phen­yl)ethane-1,2-di­yl]bis­(phenyl­diazene), for which triclinic (refcode PAGCEI; Mohamed *et al.*, 2016 ▸) and monoclinic (PAGCEI01; Mohamed *et al.*, 2016 ▸) polymorphs are known. In both polymorphs, the mol­ecules lie on inversion centres, but in PAGCEI01, the mol­ecules are subject to whole-mol­ecule disorder equivalent to configurational disorder with occupancies of 0.6021 (19) and 0.3979 (19). There are no hydrogen bonds in the crystal structure of PAGCEI, whereas the mol­ecules of PAGCEI01 are linked by C—H⋯π(arene) hydrogen bonds into complex chains, which are further linked into sheets by C— H⋯N inter­actions.

## Synthesis and crystallization   

This bis-azo dye was synthesized according to a reported method (Maharramov *et al.*, 2018 ▸; Shikhaliyev *et al.*, 2018 ▸). A 20 mL screw neck vial was charged with DMSO (10 mL), 1,3-bis­[(*E*)-(2-phenyl­hydrazineyl­idene)meth­yl]benzene (628 mg, 2 mmol), tetra­methylethyl­enedi­amine (TMEDA) (581 mg, 5 mmol), CuCl (3 mg, 0.03 mmol) and CCl_4_ (20 mmol, 10 equiv). After 1–3 h (until TLC analysis showed complete consumption of the corresponding Schiff base), the reaction mixture was poured into a 0.01 *M* solution of HCl (100 mL, pH = 2–3), and extracted with di­chloro­methane (3 × 20 mL). The combined organic phase was washed with water (3 × 50 mL), brine (30 mL), dried over anhydrous Na_2_SO_4_ and concentrated *in vacuo* using a rotary evaporator. The residue was purified by column chromatography on silica gel using appropriate mixtures of hexane and di­chloro­methane (3/1–1/1). Crystals suitable for X-ray analysis were obtained by slow evaporation of a di­chloro­methane solution. Orange solid (50%); mp 402 K. Analysis calculated for C_22_H_14_Cl_4_N_4_ (*M* = 476.18): C 55.49, H 2.96, N 11.77; found: C 55.45, H 2.94, N 11.70%. ^1^H NMR (300 MHz, CDCl_3_) *δ* 6.58–8.02 (14H, Ar). ^13^C NMR (75MHz, CDCl_3_) δ 121.8, 122.15, 124.83, 126.28, 127.32, 128.04, 128.95, 130.09, 133.12, 139.07. ESI–MS: *m*/*z*: 477.32 [*M* + H]^+^.

## Refinement   

Crystal data, data collection and structure refinement details are summarized in Table 4 ▸. All H atoms were positioned geometrically and refined using a riding model, with C—H = 0.93 Å, and with *U*_iso_(H) = 1.2*U*_eq_ (C). Owing to poor agreement between observed and calculated intensities, six outliers ( 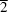 16 2, 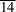 1 12, 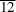 1 13, 8 14 1, 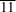 2 13 and 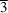 16 1) were omitted in the final cycles of refinement.

## Supplementary Material

Crystal structure: contains datablock(s) I. DOI: 10.1107/S2056989021007192/yk2154sup1.cif

Structure factors: contains datablock(s) I. DOI: 10.1107/S2056989021007192/yk2154Isup2.hkl

Supporting information file. DOI: 10.1107/S2056989021007192/yk2154Isup3.cml

CCDC reference: 1987627

Additional supporting information:  crystallographic information; 3D view; checkCIF report

## Figures and Tables

**Figure 3:**
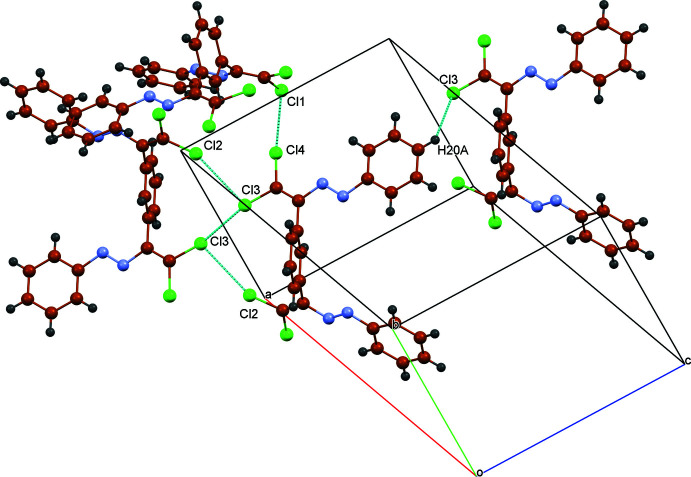
A fragment of the mol­ecular packing showing the Cl⋯Cl and Cl—H inter­actions.

**Table 1:** C—H⋯π inter­actions (Å, °) *Cg*3 is the centroid of the C17–C22 ring.

*D*—H⋯*A*	*D*—H	H⋯*A*	*D*⋯*A*	*D*—H⋯*A*
C12—H12*A*⋯*Cg*3^i^	0.93	2.72	3.610 (3)	162

Symmetry code: (i) x, -y+{\script{3\over 2}}, z-{\script{1\over 2}}.

**Table 2:** Inter­molecular contacts (Å) in the title structure

Contact	Distance	Symmetry operation
Cl4⋯*Cg*3	1.709 (2)	2 − *x*, 1 − *y*, 1 − *z*
Cl1⋯Cl4	3.4325 (12)	2 − *x*, −{1\over 2} + *y*, {1\over 2} − *z*
Cl3⋯Cl2	3.5171 (13)	2 − *x*, 1 − *y*, −*z*
H14*A*⋯C7	2.97	*x*, {1\over 2} − *y*, −{1\over 2} + *z*
Cl3⋯H20*A*	3.10	*x*, *y*, − 1 + *z*
H13*A*⋯C4	2.95	1 − *x*, 1 − *y*, −*z*
H7*A*⋯H4*A*	2.43	1 − *x*, −{1\over 2} + *y*, {1\over 2} − *z*
H12*A*⋯C21	2.92	*x*, {3\over 2} − *y*, −{1\over 2} + *z*
H8*A*⋯H7*A*	2.54	1 − *x*, −*y*, −*z*

**Table 3:** Percentage contributions of inter­atomic contacts to the Hirshfeld surface for the title compound

Contact	Percentage contribution
H⋯H	30.4
C⋯H/H⋯C	20.4
Cl⋯H/H⋯Cl	19.4
Cl⋯Cl	7.8
Cl⋯C/C⋯Cl	7.3
Cl⋯N/N⋯Cl	5.9
N⋯H/H⋯N	5.6
C⋯C	1.8
N⋯C/C⋯N	1.2
N⋯N	0.2

**Table 4:** Experimental details

Crystal data	
Chemical formula	C_22_H_14_Cl_4_N_4_
*M*_r_	476.17
Crystal system, space group	Monoclinic, *P*2_1_/*c*
Temperature (K)	296
*a*, *b*, *c* (Å)	16.0289 (10), 13.1213 (8), 11.1286 (7)
β (°)	108.073 (2)
*V* (Å^3^)	2225.1 (2)
*Z*	4
Radiation type	Mo *K*α
μ (mm^−1^)	0.55
Crystal size (mm)	0.44 × 0.26 × 0.12

Data collection	
Diffractometer	Bruker APEXII CCD
Absorption correction	Multi-scan (*SADABS*; Krause *et al.*, 2015 ▸)
*T*_min_, *T*_max_	0.621, 0.745
No. of measured, independent and observed [*I* > 2σ(*I*)] reflections	24362, 4399, 3193
*R*_int_	0.044
(sin θ/λ)_max_ (Å^−1^)	0.626

Refinement	
*R*[*F* ^2^ > 2σ(*F* ^2^)], *wR*(*F* ^2^), *S*	0.046, 0.112, 1.01
No. of reflections	4399
No. of parameters	271
H-atom treatment	H-atom parameters constrained
Δρ_max_, Δρ_min_ (e Å^−3^)	0.24, −0.25

Computer programs: *APEX3* and *SAINT* (Bruker, 2007 ▸), *SHELXT2016/6* (Sheldrick, 2015*a* ▸), *SHELXL2016/6* (Sheldrick, 2015*b* ▸), *ORTEP-3 for Windows* (Farrugia, 2012 ▸) and *PLATON* (Spek, 2020 ▸).

## supplementary crystallographic information

### Crystal data

**Table:**

C_22_H_14_Cl_4_N_4_	*F*(000) = 968
*M**_r_* = 476.17	*D*_x_ = 1.421 Mg m^−^^3^
Monoclinic, *P*2_1_/*c*	Mo *K*α radiation, λ = 0.71073 Å
*a* = 16.0289 (10) Å	Cell parameters from 6439 reflections
*b* = 13.1213 (8) Å	θ = 2.5–26.4°
*c* = 11.1286 (7) Å	µ = 0.55 mm^−^^1^
β = 108.073 (2)°	*T* = 296 K
*V* = 2225.1 (2) Å^3^	Prism, colourless
*Z* = 4	0.44 × 0.26 × 0.12 mm

### Data collection

**Table:**

Bruker APEXII CCD diffractometer	3193 reflections with *I* > 2σ(*I*)
φ and ω scans	*R*_int_ = 0.044
Absorption correction: multi-scan (SADABS; Krause *et al.*, 2015)	θ_max_ = 26.4°, θ_min_ = 2.1°
*T*_min_ = 0.621, *T*_max_ = 0.745	*h* = −20→20
24362 measured reflections	*k* = −16→16
4399 independent reflections	*l* = −13→13

### Refinement

**Table:**

Refinement on *F*^2^	Primary atom site location: difference Fourier map
Least-squares matrix: full	Secondary atom site location: difference Fourier map
*R*[*F*^2^ > 2σ(*F*^2^)] = 0.046	Hydrogen site location: inferred from neighbouring sites
*wR*(*F*^2^) = 0.112	H-atom parameters constrained
*S* = 1.01	*w* = 1/[σ^2^(*F*_o_^2^) + (0.0456*P*)^2^ + 1.0697*P*] where *P* = (*F*_o_^2^ + 2*F*_c_^2^)/3
4399 reflections	(Δ/σ)_max_ = 0.001
271 parameters	Δρ_max_ = 0.24 e Å^−^^3^
0 restraints	Δρ_min_ = −0.25 e Å^−^^3^

### Special details

**Table:**

Geometry. All esds (except the esd in the dihedral angle between two l.s. planes) are estimated using the full covariance matrix. The cell esds are taken into account individually in the estimation of esds in distances, angles and torsion angles; correlations between esds in cell parameters are only used when they are defined by crystal symmetry. An approximate (isotropic) treatment of cell esds is used for estimating esds involving l.s. planes.

### Fractional atomic coordinates and isotropic or equivalent isotropic displacement parameters (Å^2^)

**Table:**

	*x*	*y*	*z*	*U*_iso_*/*U*_eq_	
Cl1	0.74226 (5)	0.14141 (5)	−0.10025 (7)	0.0592 (2)	
Cl2	0.81687 (6)	0.33515 (7)	−0.12359 (8)	0.0777 (3)	
Cl3	0.97228 (5)	0.57996 (7)	0.11006 (7)	0.0736 (3)	
Cl4	1.05825 (5)	0.63410 (7)	0.36813 (8)	0.0762 (3)	
N1	0.65092 (13)	0.24501 (16)	0.05084 (19)	0.0467 (5)	
N2	0.60887 (13)	0.28678 (16)	0.11510 (19)	0.0488 (5)	
N3	0.88872 (14)	0.60214 (16)	0.40656 (19)	0.0469 (5)	
N4	0.81623 (14)	0.58883 (16)	0.42594 (18)	0.0468 (5)	
C1	0.74926 (16)	0.2676 (2)	−0.0611 (2)	0.0472 (6)	
C2	0.70658 (15)	0.31073 (19)	0.0111 (2)	0.0435 (6)	
C3	0.55342 (16)	0.2175 (2)	0.1536 (2)	0.0480 (6)	
C4	0.5054 (2)	0.2569 (3)	0.2249 (3)	0.0698 (8)	
H4A	0.510609	0.325648	0.246399	0.084*	
C5	0.4494 (2)	0.1959 (3)	0.2652 (3)	0.0830 (10)	
H5A	0.417287	0.223700	0.313982	0.100*	
C6	0.44075 (19)	0.0957 (3)	0.2343 (3)	0.0721 (9)	
H6A	0.402268	0.054889	0.260581	0.086*	
C7	0.4887 (2)	0.0553 (3)	0.1645 (3)	0.0730 (9)	
H7A	0.483059	−0.013532	0.143736	0.088*	
C8	0.5458 (2)	0.1151 (2)	0.1240 (3)	0.0641 (8)	
H8A	0.578832	0.086471	0.077119	0.077*	
C9	0.71822 (15)	0.41985 (18)	0.0494 (2)	0.0419 (5)	
C10	0.79331 (15)	0.45017 (18)	0.1441 (2)	0.0409 (5)	
H10A	0.835438	0.401942	0.183488	0.049*	
C11	0.80614 (15)	0.55110 (18)	0.1805 (2)	0.0386 (5)	
C12	0.74374 (16)	0.62289 (19)	0.1206 (2)	0.0465 (6)	
H12A	0.751924	0.691101	0.144018	0.056*	
C13	0.66925 (17)	0.5928 (2)	0.0259 (2)	0.0545 (7)	
H13A	0.627449	0.641110	−0.014303	0.065*	
C14	0.65639 (16)	0.4924 (2)	−0.0093 (2)	0.0510 (6)	
H14A	0.605927	0.472971	−0.072944	0.061*	
C15	0.96300 (16)	0.5965 (2)	0.2578 (2)	0.0498 (6)	
C16	0.88676 (15)	0.58312 (18)	0.2816 (2)	0.0418 (5)	
C17	0.82000 (18)	0.60524 (19)	0.5545 (2)	0.0477 (6)	
C18	0.89313 (19)	0.6384 (2)	0.6491 (2)	0.0571 (7)	
H18A	0.944637	0.653856	0.631148	0.068*	
C19	0.8890 (2)	0.6485 (2)	0.7705 (3)	0.0670 (8)	
H19A	0.938099	0.670842	0.834584	0.080*	
C20	0.8130 (3)	0.6258 (2)	0.7979 (3)	0.0717 (9)	
H20A	0.810894	0.632115	0.880182	0.086*	
C21	0.7409 (2)	0.5940 (2)	0.7040 (3)	0.0699 (8)	
H21A	0.689496	0.578686	0.722387	0.084*	
C22	0.74340 (19)	0.5843 (2)	0.5820 (3)	0.0589 (7)	
H22A	0.693555	0.563666	0.518131	0.071*	

### Atomic displacement parameters (Å^2^)

**Table:**

	*U*^11^	*U*^22^	*U*^33^	*U*^12^	*U*^13^	*U*^23^
Cl1	0.0678 (4)	0.0510 (4)	0.0588 (4)	0.0018 (3)	0.0197 (3)	−0.0113 (3)
Cl2	0.0910 (6)	0.0744 (5)	0.0899 (6)	−0.0174 (4)	0.0603 (5)	−0.0081 (4)
Cl3	0.0575 (4)	0.1103 (7)	0.0610 (5)	−0.0054 (4)	0.0302 (3)	−0.0060 (4)
Cl4	0.0446 (4)	0.0956 (6)	0.0776 (5)	−0.0107 (4)	0.0032 (3)	−0.0124 (4)
N1	0.0497 (11)	0.0487 (13)	0.0435 (12)	−0.0082 (10)	0.0171 (10)	−0.0042 (10)
N2	0.0497 (12)	0.0525 (13)	0.0460 (12)	−0.0077 (10)	0.0173 (10)	−0.0055 (10)
N3	0.0532 (12)	0.0440 (12)	0.0414 (11)	−0.0046 (10)	0.0115 (10)	−0.0043 (9)
N4	0.0557 (12)	0.0452 (12)	0.0397 (11)	−0.0045 (10)	0.0148 (10)	−0.0026 (9)
C1	0.0491 (14)	0.0484 (15)	0.0438 (14)	−0.0029 (12)	0.0141 (11)	−0.0023 (11)
C2	0.0440 (13)	0.0460 (14)	0.0384 (13)	−0.0071 (11)	0.0095 (10)	−0.0037 (11)
C3	0.0451 (13)	0.0547 (16)	0.0432 (14)	−0.0065 (12)	0.0122 (11)	−0.0016 (12)
C4	0.0744 (19)	0.065 (2)	0.082 (2)	−0.0045 (16)	0.0424 (18)	−0.0088 (16)
C5	0.074 (2)	0.099 (3)	0.095 (3)	−0.006 (2)	0.053 (2)	−0.004 (2)
C6	0.0568 (17)	0.093 (3)	0.067 (2)	−0.0182 (17)	0.0208 (15)	0.0087 (18)
C7	0.090 (2)	0.062 (2)	0.071 (2)	−0.0208 (17)	0.0303 (18)	0.0000 (16)
C8	0.0746 (19)	0.0585 (19)	0.0672 (19)	−0.0088 (15)	0.0335 (16)	−0.0058 (15)
C9	0.0448 (12)	0.0448 (14)	0.0382 (12)	−0.0062 (11)	0.0159 (10)	−0.0036 (11)
C10	0.0415 (12)	0.0418 (14)	0.0399 (13)	−0.0003 (10)	0.0136 (10)	0.0027 (10)
C11	0.0426 (12)	0.0414 (14)	0.0342 (12)	−0.0043 (10)	0.0155 (10)	−0.0009 (10)
C12	0.0528 (14)	0.0410 (14)	0.0471 (14)	0.0010 (11)	0.0177 (12)	−0.0023 (11)
C13	0.0525 (15)	0.0534 (17)	0.0523 (16)	0.0110 (13)	0.0085 (12)	0.0036 (13)
C14	0.0461 (13)	0.0591 (17)	0.0422 (14)	−0.0033 (12)	0.0056 (11)	−0.0045 (12)
C15	0.0432 (13)	0.0541 (16)	0.0499 (15)	−0.0022 (12)	0.0111 (11)	−0.0041 (12)
C16	0.0454 (13)	0.0371 (13)	0.0419 (13)	−0.0019 (10)	0.0117 (10)	−0.0017 (10)
C17	0.0658 (16)	0.0389 (14)	0.0384 (13)	−0.0005 (12)	0.0160 (12)	−0.0020 (11)
C18	0.0664 (17)	0.0538 (17)	0.0474 (15)	−0.0019 (14)	0.0125 (13)	−0.0034 (13)
C19	0.090 (2)	0.0604 (19)	0.0432 (16)	−0.0001 (17)	0.0098 (15)	−0.0027 (13)
C20	0.112 (3)	0.062 (2)	0.0460 (17)	0.0052 (18)	0.0309 (18)	0.0006 (14)
C21	0.089 (2)	0.071 (2)	0.0593 (19)	−0.0048 (18)	0.0366 (17)	−0.0004 (16)
C22	0.0701 (18)	0.0583 (18)	0.0514 (16)	−0.0084 (14)	0.0233 (14)	−0.0052 (13)

### Geometric parameters (Å, º)

**Table:**

Cl1—C1	1.707 (3)	C9—C14	1.383 (3)
Cl2—C1	1.706 (3)	C9—C10	1.389 (3)
Cl3—C15	1.710 (3)	C10—C11	1.381 (3)
Cl4—C15	1.709 (2)	C10—H10A	0.9300
N1—N2	1.251 (3)	C11—C12	1.385 (3)
N1—C2	1.407 (3)	C11—C16	1.487 (3)
N2—C3	1.427 (3)	C12—C13	1.383 (3)
N3—N4	1.258 (3)	C12—H12A	0.9300
N3—C16	1.404 (3)	C13—C14	1.372 (4)
N4—C17	1.430 (3)	C13—H13A	0.9300
C1—C2	1.333 (3)	C14—H14A	0.9300
C2—C9	1.489 (3)	C15—C16	1.340 (3)
C3—C4	1.367 (4)	C17—C18	1.381 (4)
C3—C8	1.380 (4)	C17—C22	1.382 (4)
C4—C5	1.378 (4)	C18—C19	1.380 (4)
C4—H4A	0.9300	C18—H18A	0.9300
C5—C6	1.356 (5)	C19—C20	1.378 (4)
C5—H5A	0.9300	C19—H19A	0.9300
C6—C7	1.358 (4)	C20—C21	1.361 (4)
C6—H6A	0.9300	C20—H20A	0.9300
C7—C8	1.383 (4)	C21—C22	1.376 (4)
C7—H7A	0.9300	C21—H21A	0.9300
C8—H8A	0.9300	C22—H22A	0.9300
			
N2—N1—C2	114.7 (2)	C10—C11—C16	120.5 (2)
N1—N2—C3	112.9 (2)	C12—C11—C16	120.0 (2)
N4—N3—C16	114.0 (2)	C13—C12—C11	119.8 (2)
N3—N4—C17	113.3 (2)	C13—C12—H12A	120.1
C2—C1—Cl2	122.4 (2)	C11—C12—H12A	120.1
C2—C1—Cl1	124.1 (2)	C14—C13—C12	120.6 (2)
Cl2—C1—Cl1	113.55 (15)	C14—C13—H13A	119.7
C1—C2—N1	115.1 (2)	C12—C13—H13A	119.7
C1—C2—C9	122.6 (2)	C13—C14—C9	120.2 (2)
N1—C2—C9	122.3 (2)	C13—C14—H14A	119.9
C4—C3—C8	118.9 (3)	C9—C14—H14A	119.9
C4—C3—N2	116.6 (3)	C16—C15—Cl4	124.2 (2)
C8—C3—N2	124.5 (2)	C16—C15—Cl3	122.0 (2)
C3—C4—C5	120.7 (3)	Cl4—C15—Cl3	113.76 (15)
C3—C4—H4A	119.7	C15—C16—N3	115.6 (2)
C5—C4—H4A	119.7	C15—C16—C11	121.3 (2)
C6—C5—C4	120.4 (3)	N3—C16—C11	123.2 (2)
C6—C5—H5A	119.8	C18—C17—C22	119.7 (3)
C4—C5—H5A	119.8	C18—C17—N4	125.0 (3)
C5—C6—C7	119.5 (3)	C22—C17—N4	115.3 (2)
C5—C6—H6A	120.2	C19—C18—C17	119.3 (3)
C7—C6—H6A	120.2	C19—C18—H18A	120.3
C6—C7—C8	121.0 (3)	C17—C18—H18A	120.3
C6—C7—H7A	119.5	C20—C19—C18	120.7 (3)
C8—C7—H7A	119.5	C20—C19—H19A	119.7
C3—C8—C7	119.5 (3)	C18—C19—H19A	119.7
C3—C8—H8A	120.2	C21—C20—C19	119.7 (3)
C7—C8—H8A	120.2	C21—C20—H20A	120.2
C14—C9—C10	119.1 (2)	C19—C20—H20A	120.2
C14—C9—C2	121.2 (2)	C20—C21—C22	120.5 (3)
C10—C9—C2	119.6 (2)	C20—C21—H21A	119.7
C11—C10—C9	120.8 (2)	C22—C21—H21A	119.7
C11—C10—H10A	119.6	C21—C22—C17	120.0 (3)
C9—C10—H10A	119.6	C21—C22—H22A	120.0
C10—C11—C12	119.4 (2)	C17—C22—H22A	120.0
			
C2—N1—N2—C3	−179.88 (19)	C16—C11—C12—C13	179.5 (2)
C16—N3—N4—C17	178.1 (2)	C11—C12—C13—C14	0.1 (4)
Cl1—C1—C2—N1	−2.4 (3)	C12—C13—C14—C9	−0.2 (4)
Cl2—C1—C2—C9	−3.6 (3)	C10—C9—C14—C13	−0.1 (4)
Cl1—C1—C2—C9	176.39 (18)	C2—C9—C14—C13	−178.8 (2)
N2—N1—C2—C1	−177.7 (2)	Cl4—C15—C16—N3	0.0 (3)
N2—N1—C2—C9	3.5 (3)	Cl3—C15—C16—N3	−178.32 (18)
N1—N2—C3—C4	179.7 (2)	Cl4—C15—C16—C11	179.46 (19)
N1—N2—C3—C8	−0.1 (4)	Cl3—C15—C16—C11	1.2 (4)
C8—C3—C4—C5	−0.9 (5)	N4—N3—C16—C15	−179.9 (2)
N2—C3—C4—C5	179.3 (3)	N4—N3—C16—C11	0.6 (3)
C3—C4—C5—C6	−0.3 (5)	C10—C11—C16—C15	79.5 (3)
C4—C5—C6—C7	0.9 (5)	C12—C11—C16—C15	−99.6 (3)
C5—C6—C7—C8	−0.4 (5)	C10—C11—C16—N3	−101.1 (3)
C4—C3—C8—C7	1.4 (4)	C12—C11—C16—N3	79.9 (3)
N2—C3—C8—C7	−178.8 (3)	N3—N4—C17—C18	3.8 (4)
C6—C7—C8—C3	−0.7 (5)	N3—N4—C17—C22	−175.5 (2)
C1—C2—C9—C14	102.4 (3)	C22—C17—C18—C19	1.1 (4)
N1—C2—C9—C14	−79.0 (3)	N4—C17—C18—C19	−178.1 (2)
C1—C2—C9—C10	−76.3 (3)	C17—C18—C19—C20	0.0 (4)
N1—C2—C9—C10	102.4 (3)	C18—C19—C20—C21	−0.6 (5)
C14—C9—C10—C11	0.6 (3)	C19—C20—C21—C22	0.1 (5)
C2—C9—C10—C11	179.3 (2)	C20—C21—C22—C17	1.1 (5)
C9—C10—C11—C12	−0.8 (3)	C18—C17—C22—C21	−1.7 (4)
C9—C10—C11—C16	−179.8 (2)	N4—C17—C22—C21	177.6 (3)
C10—C11—C12—C13	0.4 (4)		

### Hydrogen-bond geometry (Å, º)

*Cg*3 is the centroid of the C17–C22 ring.

**Table:**

*D*—H···*A*	*D*—H	H···*A*	*D*···*A*	*D*—H···*A*
C12—H12*A*···*Cg*3^i^	0.93	2.72	3.610 (3)	162

Symmetry code: (i) *x*, −*y*+3/2, *z*−1/2.
